# Assessing the Ovarian Accessory Glands to Determine the Parity of *Phlebotomus papatasi*, Vector of Zoonotic Cutaneous Leishmaniasis, under Laboratory Condition

**Published:** 2017-03-14

**Authors:** Mahboubeh Fatemi, Mohammad Reza Yaghoobi-Ershadi, Mehdi Mohebali, Zahra Saeidi, Arshad Veysi, Ali Khamesipour, Amir Ahmad Akhavan

**Affiliations:** 1 Department of Medical Entomology and Vector Control, School of Public Health, Tehran University of Medical Sciences, Tehran, Iran; 2 Department of Medical Parasitology, School of Public Health, Tehran University of Medical Sciences, Tehran, Iran; 3 Center for Research and Training in Skin Diseases and Leprosy, Tehran University of Medical Sciences, Tehran, Iran

**Keywords:** *Phlebotomus papatasi*, Accessory glands, Parous, Nulliparous, Parity

## Abstract

**Background:**

Zoonotic cutaneous leishmaniasis (ZCL) is a neglected tropical disease prevailed in many rural areas of 17 out of 31 provinces in Iran. The main vector of the disease is *Phlebotomus papatasi* and the causative agent is *Leishmania major* in ZCL foci of Iran. In the current study we investigated the validity of accessory glands secretions as an indicator to recognize parous from nulliparous *Ph. papatasi* females under laboratory conditions.

**Methods:**

Over 235 laboratory-reared females of *Ph. papatasi* were dissected in 6 groups including: newly emerged, one hour, one day, two days, and three days after blood feeding and also after oviposition under stereo microscope for their parity in 2014–2015.

**Results:**

Transparent glands were compatible with nulliparous only in newly emerged sand flies. In sand flies dissected after oviposition, accessory glands were rather large as a result of oviposition though they were transparent.

**Conclusion:**

The accessory glands secretions could not be as an indicator for distinguishing parous from nulliparous of *Ph. papatasi* females.

## Introduction

Phlebotominae sand flies are the vectors of different kinds of leishmaniasis and papatasi fever in the world (Dejeux 1991). Different forms of leishmaniasis have been recorded from 98 countries and 350 million people are at risk of the disease worldwide (WHO 2010). Zoonotic cutaneous leishmaniasis (ZCL) is the most common form of cutaneous leishmaniasis. Annually around 20000 new cases of leishmaniasis are reported in Iran, which more than 80% of the cases are ZCL form (Shirzadi 2010). *Rhombomys opimus* (great gerbil) is the major reservoir host of the disease in central and north-east parts of Iran. *Tatera indica* and *Meriones hurrianae* are the main reservoirs of the disease in south-west and southeast parts of Iran respectively (Mohebali et al. 2004, Akhavan et al. 2010). *Meriones libycus* is also reported as the main reservoir of ZCL in some central and south parts of the country. *Leishmania major* is the causative agent of the disease in Iran (Yaghoobi-Ershadi et al. 1996). *Phlebotomus papatasi* and *Ph. caucasicus* are proven vectors of the enzootic cycle of *L. major* among gerbils and jirds but only *Ph. papatasi* is responsible for transmitting the disease to human (Yaghoobi-Ershadi et al. 1994).

In the course of disease transmission by sand fly, only those flies have laid eggs once (parous), could transmit the disease. Thus, parous sand flies play a critical role in disease spreading. The study of age composition in phlebotominae sand flies should be a fundamental part for understanding the epidemiology of the disease transmitted by them, particularly leishmaniasis. On the other hand, physiological age of phlebotomine sand flies is estimated by nulliparous and parous female recognition. Unlike mosquitoes ovariole characteristics in phlebotominae due to small size, are not useful method to determine physiological age (Lewis and Minter 1960, Lewis 1965).

So far no method has been applicable for counting the number of dilatations in sand flies (Detinova 1962). Ovarian accessory glands in sand flies are relatively large and have been used as a practical method to determine parity in several sand fly species (Adler and Theodor 1935, Lewis and Minter 1960, Lewis 1970, Takaoka 1989) but this method is not applicable for distinguishing parous females in many other species (Lewis 1965, Scorza 1968, Ready et al. 1984, Takaoka 1989).

At the current study the reliability of the ovarian accessory glands to distinguish parity of *Ph. papatasi* females was examined under laboratory conditions.

## Materials and Methods

Sand flies were collected using aspirating tubes during the active seasons from rural districts of endemic areas of Esfahan Province, Iran in 2014–2015. *Phlebotomus papatasi* colony was reared at the sand fly insectary of Medical Entomology and Vector Control Department, School of Public Health, Tehran University of Medical Sciences and the Esfahan Health Research Station.

*Phlebotomus papatasi* sand flies were reared under laboratory conditions at 26±2 °C, 70–80% relative humidity and 14: 10 h (L: D) photoperiod.

A male and a blood-fed female were transferred in to plaster-lined individual pot for oviposition (Killick-Kendrick and Killick-Kendrick 1991). From the 2^nd^ generation, the mass-rearing technique was used (Modi and Tesh 1983). To prevent fungal contamination in rearing pots, autoclaved field soil and sea sand were added into the rearing pots. Larvae food was sprinkled after 50% eggs hatching.

Larval diet is the most important factor for maintaining sand fly colony. Larvae food was prepared using the method described by Young et al. (1981) with some modifications. In brief, the larvae food consists of equal proportions of dried rabbit feces, rabbit chow and yeast. Rabbit feces and chow were ground by a grinder. A spoonful yeast dissolved in distilled water, then added for each 120-gr powder, then enough amount of distilled water added to make paste. After 2 days when fungi contamination disappeared, the mixture was spread in a thin layer inside a tray. It was allowed to dry in an upside down position. Then food was scraped from the trays and ground by a grinder. After about one week the larval food was ready to use.

**Fig. 1.**
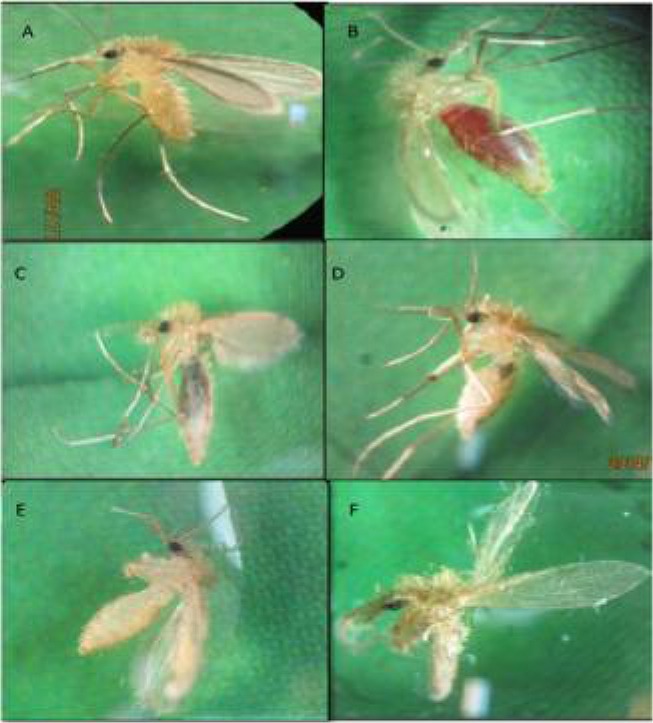
Different stages of abdominal physiology in *Phlebotomus papatasi* A) Newly emerged, B) One hour after blood feeding, C) One day after blood feeding, D) Two days after blood feeding, E) Three days after blood feeding, F) After oviposition

For checking the value of using accessory glands for distinguishing parous of *Ph. papatasi* females from nulliparous, over 235 laboratory-reared females of *Ph. papatasi* were dissected in 6 groups including: newly emerged, one hour, one day, two days and three days after blood feeding and also after oviposition under stereo microscope for its parity (Fig. 1).

Prior to dissection, sand flies were immobilized in −20 °C freezer for 5 min. Each sand fly was placed in a drop of sterile normal saline (9/1000) on a clean slide and dissected under a stereomicroscope. After releasing the ovaries and accessory glands, immediately they were examined under a stereomicroscope and light microscope to check the presence of granular secretions of the accessory glands.

## Results

A total of 235 *Ph. papatasi* females including 30 sand flies “newly emerged”, 33 sand flies “one hour after blood feeding”, 50 sand flies “one day after blood feeding”, 38 sand flies “two days after blood feeding”, 49 sand flies “three days after blood feeding and 35 sand flies “after oviposition” were dissected and compared regarding granular secretions (Table 1).

**Table 1.** Observation on accessory glands of dissected *Phlebotomus papatasi*

**Groups**	**Accessory glands secretions**				**Total**

	**Absent N (%)**		**Present N (%)**		

	**Nulliparous**	**Parous**	**Nulliparous**	**Parous**	
**Newly emerged**	30 (100)	-	-	-	30
**One hour after blood feeding**	17 (51.5)	-	16 (48.5)	-	33
**One day after blood feeding**	-	-	50 (100)	-	50
**Two days after blood feeding**	-	-	38 (100)	-	38
**Three days after blood feeding**	-	-	49 (100)	-	49
**After oviposition**	-	9 (25.71)	-	26 (74.29)	35
					235

Granular secretions in the accessory glands were not observed in nulliparous females in newly emerged sand flies. In 17 out of 33 *Ph. papatasi* dissected one hour after blood feeding, granular secretions in the accessory glands were not observed in nulliparous females as well.

**Fig. 2.**
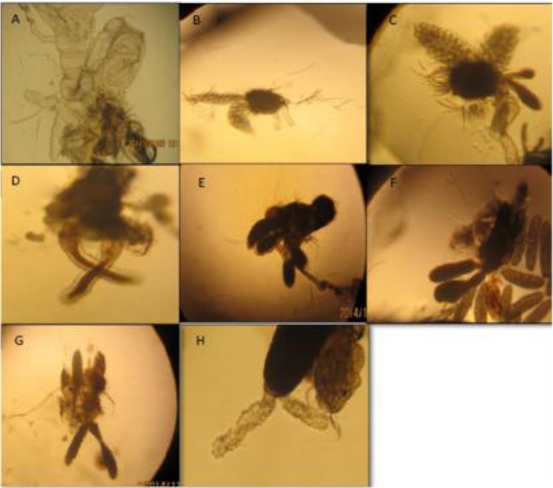
Accessory gland status of *Phlebotomus papatasi* in different stages of abdominal physiology. A) Newly emerged, B, C) One hour after blood feeding, D) One day after blood feeding, E) Two days after blood feeding, F) Three days after blood feeding, G, H) After oviposition

In contrast, third, fourth and fifth groups of sand flies which were dissected one day after blood feeding, two days after blood feeding and three days after blood feeding showed discordant relation between granular secretions and parity. Discordant relation between granular secretions and parity was also found in 9 out of 35 of the sand flies after oviposition, although accessory glands were rather large as a result of oviposition. In 26 of 35 *Ph. papatasi* sand flies which had laid eggs granular secretions in the accessory glands were observed (Fig. 2).

## Discussion

Accessory glands are convenient to determine parity of Phlebotominae sand flies because they are enough large to inspect fairly and quickly in contrast to ovarian but the results of the current study and some others show that reliability of this method depends on sand fly species and it seems to be impractical for all the sandflies. So all females with granular secretions cannot be considered as parous, because some of the blood fed nulliparous females might have secreted granules shortly after blood feeding.

Accessory glands secretions in *Ph. califoniicus*, *Ph. stewarti* and *Ph. vexator* from northern California and also in ten species from Kenya including *Se. suberecta*, *Se. clydei*, *Se. garnhami*, *Se. ingrami*, *Se. harveyi*, *Se. schwetzi*, *Se. bedfordi*, *Se. antennata*, *Ph. martini* and *Ph. guggisbergi* are a useful method of parity (Lewis and Minter 1960, Chanitois and Anderson 1967) in contrast granular secretions were seen in both parus and nuliparus females of *Lu. cruciata*, *Lu. ovallesi*, *Lu. ylephiletrix*, *Lu. panamensis* and *Lu. shannoni* (Lewis 1965).

Accessory glands of *Lu. townsedi* in Venezuela showed discordant relation between granular secretions and parity as well (Scorza et al. 1968).

Lewis (1970) reported that accessory glands secretions are a good indicator in eight species such as *Lu. antunesi*, *Lu. flaviscutellata*, *Lu. ubiquilalis*, *Lu. aragaoi*, *Lu. rorotaensis*, *Lu. infraspinosa*, *Lu. tuberculata* and *Lu. saulensis* although with small errors.

In all dissected females of *Lu. gomezi* and *Lu. shannoni* accessory glands secretions were seen. As a result, accessory glands are not useful for distinguishing parity (Hashiguchi 1987).

Takaoka et al. (1989) reported that accessory glands are a reliable sign of parity in females of *Lu. ayacuchensis*. But they are not suitable in six species including *Lu. trapidoi*, *Lu. hartmanni*, *Lu. carrerai*, *Lu. thula*, *Lu. panamensis*, *Lu. shannoni* and *Lu. gomezi.* The results of above studies show that the validity of accessory glands secretions as an indicator to recognize parous from nulliparous depends on the species of sand flies. According to the results of the current study, accessory gland secretions are not good marker for determining parus from nulliparous females of *Ph. papatasi*.

## Conclusion

This study obviously demonstrates that microscopic test of the accessory gland secretions is not reliable method for determining parus from nulliparous females of *Ph. papatasi* therefore a new method should be introduced to recognize parity status of this species.

## References

[B1] Adler S Theodor O ( 1935) I Investigations on Mediterranean Kala Azar. Viii.--further Observations on Mediterranean Sandflies. Proc R Soc Lond B Biol Sci. 116( 801): 505– 515.

[B2] Akhavan AA Yaghoobi-Ershadi MR Khamesipour A Mirhendi H Alimohammadian MH Rassi Y Arandian MH Jafari R Abdoli H Shareghi N Ghanei M Jalali-zand N ( 2010) Dynamics of *Leishmania* infection rates in *Rhombomys opimus* (Rodentia: Gerbillinae) population of an endemic focus of zoonotic cutaneous leishmaniasis in Iran. Bull Soc Pathol Exot. 103( 2): 84– 89. 20390397 10.1007/s13149-010-0044-1

[B3] Chaniotis BN Anderson JA ( 1967) Age structure, population dynamics and vector potential of *Phlebotomus* in North California. Part I: Distinguishing parous from nuliparous flies. J Med Entomol. 4: 251– 254. 6052134 10.1093/jmedent/4.3.251

[B4] Dejeux P ( 1991) Information on the epidemiology and control of the leishmaniasis, by country or territory. World Health Organ, Geneva.

[B5] Detinova TS ( 1962) Age-grouping methods in Diptera of medical importance. Wld Hlth Org. 47: 216. 13885800

[B6] Hashiguchi Y ( 1987) Studies on New world leishmaniasis and its transmission, with particular reference to Ecuador, Kyowa printing and Ltd, Kochi 174.

[B7] Killick-Kendrick M Killick-Kendrick R ( 1991) The initial establishment of sand fly colonies. Parassitologia. 33 Suppl: 315– 320. 1841223

[B8] Lewis DJ ( 1965) Internal structural features of some central American phlebotomine sandflies. Ann Trop Med Parasitol. 59: 375– 385. 5893195 10.1080/00034983.1965.11686322

[B9] Lewis DJ Lainson R Shaw JJ ( 1970) Determination of parous rates in Phlebotomine sandflies with special reference to Amazonian species. Bull Entomol Res. 60: 209– 219. 22894839 10.1017/S0007485300040736

[B10] Lewis DJ Minter DM ( 1960) Internal structural changes in some African Phlebotominae. Ann Trop Med Parasitol. 54: 351– 365. 13761696 10.1080/00034983.1960.11685997

[B11] Modi GB Tesh RB ( 1983) A simple technique for mass rearing *Lutzomyia Longipalpis* and *Phlebotomus papatasi* (Diptera: Psychodidae) in the laboratory. J Med Entomol. 20: 568– 569. 6644754 10.1093/jmedent/20.5.568

[B12] Mohebali M Javadian E Yaghoobi-Ershadi MR Akhavan AA Hajjaran H Abaei MR, ( 2004) Charactrization of leishmania infection in rodents from endemic areas of Islamic Republic of Iran. East Mediterr Health J. 10: 591– 599. 16335651

[B13] Ready PD Lainson R Wilkes TJ Killick-Kendrick R ( 1984) On the accuracy of age-grading neotropical phlebotomines by counting follicular dilatations: first laboratory experiments, using colonies of *Lutzomyia flaviscutellata* (Mangabeira) and *L. furcata* (Mangabeira) (Diptera: Psychodidae). Bull Entomol Res. 74: 641– 646.

[B14] Scorza JV Ortiz I Gomez I ( 1968) Observationes biologicas sobre algunos flebotomos de Rancho Grande (Venezuela). Acta Boil Venez. 6: 52– 65 (In Spanish).

[B15] Shirzadi M ( 2010) Guideline for control of cutaneous leishmaniasis. Department of Zoonosis, CDC, Ministry of Health and Medical Education, Tehran, Iran.

[B16] Takaoka H Gomez E Alexander J Hashiguchi Y ( 1989) Observations on the validity of the ovarian accessory glands of seven ecuadorian sand fly species (Diptera: Psychodidae) in determining their parity. Japan J Trop Med Hyg. 17( 2): 149– 155.

[B17] WHO ( 2010) Control of the leishmaniases: report of a meeting of the WHO Expert Commitee on the Control of Leishmaniases, Geneva, 22–26 March 2010.

[B18] Yaghoobi-Ershadi MR Javadian E Tahvildare-Bidruni Gh ( 1994) The isolation of *Leishmanha major* from *Phlebotomus* (*paraphleboyomus*) *caucasicus,* in Isfahan Province, Islamic Republic of Iran. Trans R Soc Trop Med Hyg. 88: 518– 519. 7992325 10.1016/0035-9203(94)90142-2

[B19] Yaghoobi-Ershadi MR Akhavan AA Mohebali M ( 1996) *Meriones libycus* and *Rhombomys opimus* (Rodentia: gerbillidae) are the main reservoir hosts in a new focus of zoonotic cutaneous leishmaniasis in Iran. Trans R Soc Trop Med Hyg. 90: 503– 504. 8944255 10.1016/s0035-9203(96)90295-3

[B20] Young DG Perkins PV Endris RG ( 1981) A larval diet for rearing phlebotomine sandflies (Diptera: Psychodidae). J Med Entomol. 18( 5): 446.

